# Strategies for Interfering With Bacterial Early Stage Biofilms

**DOI:** 10.3389/fmicb.2021.675843

**Published:** 2021-06-08

**Authors:** Jingyuan Fu, Yuning Zhang, Shiyu Lin, Wei Zhang, Gang Shu, Juchun Lin, Haohuan Li, Funeng Xu, Huaqiao Tang, Guangneng Peng, Ling Zhao, Shiqi Chen, Hualin Fu

**Affiliations:** Innovative Engineering Research Center of Veterinary Pharmaceutics, Department of Pharmacy, College of Veterinary Medicine, Sichuan Agricultural University, Chengdu, China

**Keywords:** bacteria, early stage biofilms, anti-biofilm molecules, mechanisms, quorum sensing, c-di-GMP

## Abstract

Biofilm-related bacteria show high resistance to antimicrobial treatments, posing a remarkable challenge to human health. Given bacterial dormancy and high expression of efflux pumps, persistent infections caused by mature biofilms are not easy to treat, thereby driving researchers toward the discovery of many anti-biofilm molecules that can intervene in early stage biofilms formation to inhibit further development and maturity. Compared with mature biofilms, early stage biofilms have fragile structures, vigorous metabolisms, and early attached bacteria are higher susceptibility to antimicrobials. Thus, removing biofilms at the early stage has evident advantages. Many reviews on anti-biofilm compounds that prevent biofilms formation have already been done, but most of them are based on compound classifications to introduce anti-biofilm effects. This review discusses the inhibitory effects of anti-biofilm compounds on early stage biofilms formation from the perspective of the mechanisms of action, including hindering reversible adhesion, reducing extracellular polymeric substances production, interfering in the quorum sensing, and modifying cyclic di-GMP. This information can be exploited further to help researchers in designing new molecules with anti-biofilm activity.

## Introduction and Biofilms Formation

At present, most chronic and recurrent infections are associated with biofilms, such as cystic fibrosis and urinary tract infections in humans (Lories et al., 2020; Rumbaugh and Sauer, 2020). Biofilms are defined as three-dimensional (3D) structures that microbial cells attach to a surface, or to each other and embed in self-secreted extracellular polymeric substances (EPSs). EPSs consist of exopolysaccharides, extracellular DNA (eDNA), proteins, and other minor components (Yan and Bassler, 2019; Zea et al., 2020). Once bacteria form biofilms, EPSs will act as a physical and chemical barrier to protect internal bacteria. On the one hand, EPSs enable bacteria to escape the immune system by covering bacterial surface epitopes, releasing extracellular nucleases to degrade neutrophil extracellular traps, and inducing the M2 activation of macrophages (Paharik and Horswill, 2016; Parastan et al., 2020). On the other hand, EPSs remarkable enhance bacterial resistance to antimicrobials (Karygianni et al., 2020). And the resident cells will be spread into new sites to start a new life cycle of biofilms (Girish et al., 2019). These all contribute to the refractory and persistence of biofilm infections.

Biofilms formation is described as early, middle, and late stages (Mohammed et al., 2018; Zou and Liu, 2020). Early stage biofilms consist of reversible and irreversible adhesion stages (Karygianni et al., 2020; Parastan et al., 2020). In the reversible adhesion stage, planktonic bacteria approach and attach to the surface by means of surface appendages such as flagella and pili. However, the mobility of bacteria can easily overcome the bacteria-surface interaction and return to the planktonic state. In the irreversible adhesion stage, initially attached bacteria secrete EPS to bind to the surface covalently, gradually completing the firm attachment. In the early stage, attached bacteria can also aggregate through type IV pili-mediated twitching motilities and use EPSs to adhere surface-associated cells (Rabin et al., 2015; Wang et al., 2020). Middle stage biofilms include the formation of microcolonies and colonies. With the accumulation of bacteria, secreted EPSs increase, gradually forming a layer of water film on the surface of bacteria, and microcolonies are formed. Microcolonies further develop into colonies. The late stage is the maturation and detachment of biofilms. Mature biofilms appear as a 3D network structure with compact structure and coordinated functions. After maturity, biofilms rupture, and bacteria are dispersed to planktonic forms to start a new life cycle of biofilms.

## Regulation Systems in Biofilms Formation

Given that biofilms are the main state of bacteria, the quorum sensing (QS) system and cyclic di-GMP (c-di-GMP) signaling pathway have been shown to play a pivotal role in biofilms formation for various bacterial species, such as *Pseudomonas aeruginosa* (*P. aeruginosa*), *Escherichia coli* (*E. coli*), *Staphylococcus aureus* (*S. aureus*) (Solano et al., 2014; Jenal et al., 2017).

QS is a cell-to-cell communication in which bacteria secrete and detect signal molecules. Once a threshold level is reached, specific signals are activated to coordinate pathogenic behaviors, such as biofilms formation, bioluminescence and antibiotic resistance (Stephens and Bentley, 2020). The connection between QS and biofilms formation has been widely described. In the process of biofilms formation, the QS system regulates bacterial movement, EPSs production, and virulence factor secretion (Xia et al., 2012; Lee et al., 2018), whereas motility and EPSs play an important role in the initial adhesion stage.

Another important regulation system is c-di-GMP signaling pathway. The c-di-GMP is an important second messenger molecule that exists widely in bacteria, since its initial discovery as an allosteric factor regulating cellulose biosynthesis in *Gluconacetobacter xylinus*, bacterial movement and biofilms formation regulated by the c-di-GMP have grown (Rabin et al., 2015). Diguanylate cyclases (DGCs) containing the GGDEF domain and phosphodiesterases (PDEs) containing the EAL or HD-GYP domain are, respectively, responsible for the synthesis and degradation of c-di-GMP. Generally, a high c-di-GMP content is favorable for biofilms formation. In the reversible adhesion stage, c-di-GMP inhibits the activity of ATPase. Thus, FleQ cannot regulate downstream flagellar genes, thereby preventing the synthesis of flagella. This inhibitory effect can eliminate surface dynamics to make bacteria stick to the surface stably, and promote irreversible transformation. In the irreversible adhesion stage, c-di-GMP promotes EPS production. For example, in *P. aeruginosa*, the combination of c-di-GMP with FleQ relieves the inhibitory effect of FleQ on the pel promoter and activates transcription to produce pel polysaccharides (Baraquet et al., 2012). In *E. coli*, c-di-GMP binds to the PgaCD complex, which has polysaccharide synthesis and transport activity, thereby activating glycosyltransferase activity and promoting the *N*-acetyl glucosamine (GlcNAc) production (Steiner et al., 2013). This facilitation can enhance bacterial adhesion and further contribute to early stage biofilms formation.

## Rationales for Targeting the Early Stage Biofilms

During biofilms formation, bacteria exhibit a dynamic change in drug sensitivity. In the early stage of biofilm formation (Gu et al., 2019), bacteria switch from the free-swimming phase to the surface-attached phase. The cell-surface interaction triggers some mechanically sensitive channels on cell wall opening, making the bacteria susceptible to antimicrobials. At the same time, attached cells get close to each other to initiate active cell–cell interaction. During this process, cells divide, grow, and become increasingly sensitive to antimicrobials. However, in the middle and late stages, high expression of efflux pumps, horizontal gene transfer, and EPS matrix protection can significantly reduce the antimicrobial susceptibility (Venkatesan et al., 2015; Hall and Mah, 2017). Surface-attached cells can be more sensitive to antibiotics than planktonic cells during early stage, and the 3D structures of early stage biofilms have not been formed and are characterized by fragile structures. Removing biofilms at this time has evident advantages compared with removing at the mature stage. Inhibiting the formation of early stage biofilms can prevent their development into mature biofilms, thereby reducing biofilm-related infections.

Reversible adhesion, EPSs production, QS system, and c-di-GMP levels are important factors affecting early stage biofilms development. Therefore, reducing motility and EPSs production, and inhibiting QS system and c-di-GMP pathways are effective methods to inhibit early stage biofilms formation. Biofilms prevention strategies have been studied, however, many of these strategies are based on compounds classifications to discuss the anti-biofilm effects, such as antibiotics, phytochemicals, and antimicrobial peptides. Here, we make an effort to review the inhibitory effects of anti-biofilm molecules on early stage biofilms formation from the perspective of the mechanisms of action, providing help to researchers in designing new molecules with anti-biofilm activity. At the same time, we state the disadvantages of each solution in Table 1.

**TABLE 1:** The disadvantages of anti-early stage biofilms strategies.

**Anti-early stage biofilms strategies**		**Disadvantages**
Reducing reversible adhesion	Designing antibacterial surfaces	Only applies to device-related infections
	Reducing motility	Cannot completely inhibit biofilms formation when used alone
Inhibiting EPS production	Reducing exopolysaccharides production	Antibiotics can easily lead to multidrug-resistant microbes
	Reducing extracellular protein production Reducing eDNA production	Plant-derived components are less effective in *in vivo* models; how far plant-based treatments can be applied in the near future is not clear
Inhibition of the QS system		Current QS inhibitors are only suitable for a few bacteria
Targeting c-di-GMP signaling		The rate of discovery of inhibitors has lagged behind

## Reducing Reversible Adhesion

As shown in the introduction of biofilms formation, reversible adhesion is the first step of early stage biofilms formation. In the reversible adhesion stage, bacteria have not yet secreted EPSs, and are only weakly bound to the surface. The instability of the bacteria makes it easy to be removed and return to planktonic state, hindering the development of biofilms. Reversible adhesion is affected by surface properties and bacterial surface appendages such as flagella and pili. Therefore, this section focuses on the strategy of designing antibacterial surfaces and inhibiting the biological activity of flagella and pili.

### Designing Antibacterial Surfaces

**FIGURE 1:**
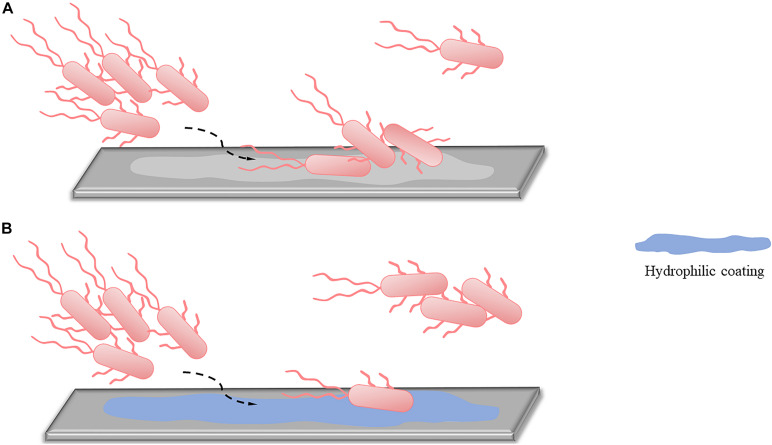
An alternative approach to reduced reversible adhesion: applying hydrophilic coatings. **(A)** Without applying hydrophilic coatings, most of the planktonic bacteria can adhere to the surface, and only a small part remains planktonic. **(B)** After using hydrophilic coatings, due to the repulsion of different charges, only a small part of planktonic bacteria can complete adhesion, while most bacteria are still in planktonic states.

Applying hydrophilic coatings on the surface is a common method to reduce bacterial adhesion in the initial stage (Figure 1). Based on the repulsive effect between different charges, the surface of bacteria has hydrophobic properties, and applying hydrophilic coatings on the surface can reduce bacterial adhesion and prevent early stage biofilms formation. Commonly used hydrophilic coatings include hyaluronic acid, polyethylene glycol (PEG), and 2-methacryloyloxy ethyl phosphorylcholine (MPC). MPC contains a phospholipid polar group similar to the cell membrane structure, which can effectively reduce protein adsorption and bacterial adhesion, thereby preventing related infections. Researchers used MPC polymers for oral bacterial biofilm infections and found that MPC polymers reduced the adhesion of *streptococci* on oral epithelial cells by 15–30% (Hirota et al., 2011). Kaneko et al. (2020) later confirmed through *in vivo* and *in vitro* experiments that applying the MPC-coated polymer to the surface of bioabsorbable suture could significantly inhibit the adhesion of *S. aureus* and reduce surgical site infections. The anti-adhesion activity of PEG is related to its molecular weight. The amount of bacterial adhesion decreases with increased molecular weight in the range of 300–20,000, and when the molecular weight is 5,000, the adhesion amount reaches the minimum (Peng et al., 2017).

In addition to hydrophilic coatings, applying antibacterial agents on the surface to kill attached bacteria directly is an effective method to inhibit reversible adhesion. Silver ion has broad-spectrum bactericidal activity and is a commonly used antibacterial coating. Stobie et al. (2009) found that a room temperature processed silver doped perfluoropolyether-urethane coating effectively reduced the adhesion of *S. aureus* on the catheter surface and prevented the formation of biofilms. Silver nanoparticles (Ag NPs) have the unique properties of nanomaterials and the antibacterial properties of silver, making them an ideal material. Researchers revealed that biosynthesized Ag NPs could reduce the adhesion of *Klebsiella pneumoniae* by 88% (Rajivgandhi et al., 2020). The antimicrobial peptide is also considered as an effective anti-biofilm coating, which shows better effect than Ag NPs in inhibiting *S. aureus* adhesion (Kang et al., 2019).

### Affecting Pili

Pili are important surface appendages that enable bacteria to realize movement and adhere to the surfaces. Thus, pili are one of the targets for removing early stage biofilms. Three types of pili are studied (De La Fuente et al., 2007; Burrows, 2012; Arita-Morioka et al., 2018): (1) Type I pili, which are best studied in *E. coli*, are a functional amyloid primarily responsible for cells’ attachment to the surface. (2) Type IV pili are the motility appendage of bacteria that mediate a kind of movement independent of flagella (i.e., twitching motility). The twitching movement plays a critical role in initial adhesion and the development of microcolonies. (3) Curli are the major extracellular surface amyloid fibers that contribute to the initial attachment to a surface and cell-to-cell contact. The drugs that act on type I pili, type IV pili, and curli are introduced in the following section.

The disperse red 15 is a hydroxyl anthraquinone compound that can interfere with FimH, an adhesion factor at the end of the type I pili of *E. coli*. After the action of disperse red 15, swimming and swarming motilities were inhibited, and the initial adhesion was reduced, resulting in a reduction in biofilm biomass by more than 50% (Miryala et al., 2020). This result indicated that disperse red 15 could better be used as surface coating to reduce bacterial adhesion and prevent biofilms formation.

Magnesium ions are essential nutrients for the formation of biofilms, which can affect bacterial adhesion and biofilm formation through direct and indirect means (Oknin et al., 2015). However, the promotion of magnesium ions on adhesion is related to its concentration. Researchers found that 0.1 and 0.5 M magnesium ions could inhibit the expression of type IV pili synthesis genes *pilV* and *pilW*, thereby reducing type IV pili formation, weakening the initial adhesion of bacteria and inhibiting early stage biofilms formation (Tang et al., 2018).

Ethylene Diamine Tetraacetic Acid (EDTA) is a metal chelating agent. Researchers found that EDTA has the strongest inhibitory effect in the early stage of biofilms formation, but has no biofilm inhibitory effect in the middle and late stages (Chang et al., 2012). EDTA plays an inhibitory role in the early stage by reducing the production of curli to inhibit cell-surface and cell–cell interactions (Chaudhary and Payasi, 2012; Gawad et al., 2017). When combined with ciprofloxacin, gentamicin, and ampicillin, EDTA could reduce the minimal inhibitory concentration of antibiotics and EDTA plus ciprofloxacin treatment had a better therapeutic effect on *P. aeruginosa* biofilms compared with the single treatment (Liu et al., 2017c).

Some bacteria also have type II and type III pili in addition to the above-mentioned pili. Type II pili are related to the pathogenic heterogeneity of *Porphyromonas gingivalis* (Inaba et al., 2008). Type III pili are an important factor in biofilms formation by *Klebsiella pneumoniae*, which may be because the pili make the surface of *K. pneumoniae* hydrophobic, thereby promoting bacterial adhesion (Martino et al., 2003). The inhibitors of these two pili are still under investigation.

### Affecting Flagella

The role of flagellar motility and surface adhesion in biofilms formation has been reported in many studies. Flagella are believed to promote bacterial adhesion (Duan et al., 2013): (1) Flagella-mediated motility makes planktonic bacteria move toward target sites, promoting subsequent adherence. (2) Flagella-mediated motility allows bacteria to overcome surface tension and reach the surface. (3) Flagella act as bacterial adhesins to help bacteria attach to the surface. Therefore, the strategy with flagella as target is also an effective method to inhibit early stage biofilms formation.

Agaric acid is a fatty acid naturally produced by certain fungi, such as *Polyporus*, and can be used as anhidrotic to treat tuberculosis patients with extreme sweating (Bacchi et al., 1969). Studies on *Salmonella* showed that the formation of biofilms was significantly inhibited after the action of agaric acid because it significantly reduced the expression of flagellar rotation genes. These phenomena inhibit the swimming ability and make bacteria stay in the planktonic stage, thereby preventing initial attachment and reducing early stage biofilms formation (Lories et al., 2020).

## Inhibiting EPS Production

**FIGURE 2:**
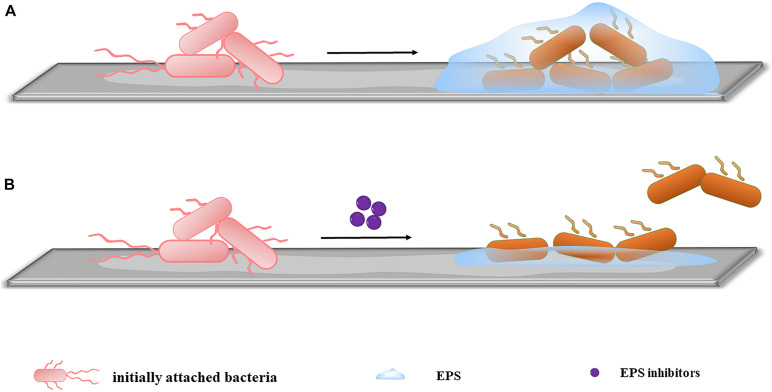
Inhibiting EPS production. **(A)** When no inhibitors are added, initially attached bacteria bind firmly to the surface by secreting EPS, completing early-stage biofilm formation. **(B)** After the addition of inhibitors, the EPS secreted by initially attached bacteria is significantly reduced. The irreversible adhesion ability of the bacteria decreases, and some bacteria return to planktonic states, inhibiting early-stage biofilm formation.

Extracellular polymeric substances mediate the irreversible adhesion stage. EPSs allow the long-term attachment of bacteria to surfaces and promote cohesion among bacterial cells, leading to the development of early stage biofilms (Flemming and Wingender, 2010; Karygianni et al., 2020). Therefore, inhibiting EPS production to reduce irreversible adhesion has become an important target (Figure 2). According to matrix components, extracellular anti-EPS molecules fall into three categories: (1) molecules acting on exopolysaccharides, (2) molecules acting on proteins, and (3) molecules acting on eDNA. These molecules destroy the structural integrity of EPS, facilitating the passage of attached bacteria into their vulnerable, planktonic state, and reduce the early stage biofilms formation. The corresponding drugs are described below.

### Reducing Exopolysaccharides Production

Among the components of EPS, the exopolysaccharides are the most abundant and the most thoroughly studied component. Different bacteria have different compositions of exopolysaccharides, which are all considered as fundamental matrix for the adhesion and architectural stability of EPS (Sharma et al., 2016; Parai et al., 2020). As molecular glue, the exopolysaccharides allow the initial adhesion of planktonic bacteria on the surface and can fix the bacterial population to increase cell density (Flemming, 2016), becoming an important target in the early stage biofilms removal strategy. At present, most drugs exert their early inhibitory effects by affecting exopolysaccharides and can be divided into chemical drugs, natural components, and probiotics.

#### Classic Antibiotics

Ambroxol (ABX) is one of the most widely used expectorant drugs. Previous studies showed that ABX could inhibit biofilms at various stages when used alone (Cataldi et al., 2014) and could enhance the killing effect of *Staphylococcus epidermidis* biofilms in combination with vancomycin (Zhang et al., 2015). It is presumed that ABX reduces the synthesis of exopolysaccharides and prevents bacterial adhesion to achieve its effect, which was later confirmed in *P. aeruginosa*. After the addition of ABX, the content of alginate decreased (Cheng et al., 2015). The expression of *algD* gene encoding the rate-limiting enzyme GMD for alginate synthesis was down-regulated. *algR* and *algU* genes, which promote synthesis, were also down-regulated whereas the *mucA* gene, which inhibits *algD* expression, was increased. These results showed that ABX influences early stage biofilms by inhibiting the synthesis of exopolysaccharides.

Antibiotics are the most used drug to treat biofilm-related infections. In this article, we take ceftazidime as an example. Ceftazidime is a β-lactam antibiotic that binds to penicillin-binding proteins to affect cell wall synthesis and reduce bacterial growth. Ceftazidime could inhibit the formation of biofilms of *Proteus mirabilis* and *E. coli* (Kwiecińska-Piróg et al., 2013; Sun et al., 2020). In studies about *P. aeruginosa* (Otani et al., 2018), ceftazidime significantly down-regulated the expression of exopolysaccharides Pel- and Psl-related genes *pelA* and *pslA* to impair the structural integrity of EPS, leading to decreased adhesion and inhibited biofilms formation. In addition, the twitching motility was suppressed. The twitching motility is mediated by type IV pili and is associated with adhesion. Ceftazidime decreased the mRNA level of *lecB* gene, which is necessary for motility, but had no effect on the transcription of the *pil* that encodes pilus components, thus weakening the expression of protein PilHIJK in twitching-related regulatory pathways, restricting twitching capacity, and affecting adhesion.

#### Plant-Derived Components

Biofilm-growing bacteria are known to show 10 to 1,000-time-increased resistance to antibiotics than planktonic microorganisms (Dosler and Karaaslan, 2014). The low efficiency of conventional doses of antibiotics and *in vivo* toxicity of high doses drive researchers toward the discovery of plant-derived anti-biofilm molecules. Compared with antibiotics, plant-derived anti-biofilm molecules have the advantages of wide sources, high safety, and low drug resistance, having great potential in controlling biofilms formation. Extracts of *Hymenocallis littoralis* leaves and tannic acid, the component of *Rhus chinensis* Mill, could inhibit the formation of *S. aureus* and *P. aeruginosa* biofilms, respectively (Trentin et al., 2013; Nadaf et al., 2018). Some plant components have been found to inhibit biofilms by inhibiting the initial adhesion of bacteria. Here are some examples.

Thymol is naturally found in the seeds of thyme and oregano, and has a broad-spectrum antimicrobial effect. Studies confirmed that thymol can inhibit the biofilms formation of *Candida tropicalis* and *Enterococcus faecalis* (Veras et al., 2014; Chatrath et al., 2019). In studies about *P. aeruginosa* and *Candida albicans* (El et al., 2011; Jafri and Ahmad, 2020), thymol is speculated to affect early stage biofilms by inhibiting adhesion. Thymol can significantly reduce the expression of the *pga* gene that codes the PGA exopolysaccharide (Wang et al., 2017), the main component of the *Actinobacillus pleuropneumoniae* biofilm matrix. This phenomenon causes changes in EPS structural components and weakens the irreversible adhesion of bacteria, which is easy to fall of the surface, thereby reducing the early stage biofilms formation. A study about *Enterobacter cloacae* showed that thymol could also inhibit the curli fimbriae *csgABCEFG* to affect initial adhesion in addition to affecting exopolysaccharide synthesis genes (Liu et al., 2020a).

Artesunate (AS), a water-soluble derivative of Artemisinin, is used in the treatment of malaria and possesses anti-cancer and anti-inflammatory activities (Kong et al., 2019). In recent years, the combination of AS and tested antibiotics can significantly enhance the antibacterial activity of antibiotics (Cremer et al., 2015). In the study with *P. aeruginosa*, Bao et al. (2019) found that using AS alone could reduce the production of exopolysaccharides, thus playing an inhibitory role in the early stage of biofilms formation. In the experiment, a concentration that had no effect on planktonic bacteria was used. In the early stage biofilms formation process, the Fenton reaction between AS and Fe(II) produces reactive oxygen species and carbon-centered radicals. No effect on bacteria is observed, but the corresponding decrease in Fe(II) increases the synthesis of rhamnolipids, competitively inhibits the precursors of polysaccharides, and reduces the production of Psl (Yu S. et al., 2016). The reduction in Psl has a negative effect on irreversible adhesion, thus inhibiting the formation of early stage biofilms. Twitching motility can promote the formation of microcolonies, which is beneficial to biofilms formation. However, excessive twitching movement has a negative effect on the formation of biofilms (Patriquin et al., 2008). Experimental results showed that AS promoted the twitching motility of *P. aeruginosa* PAO1 in a concentration-dependent manner, resulting in the difficult aggregation and attachment of bacteria and inhibited the formation of biofilms.

Phenyllactic acid (PLA) is an organic acid that exists in many plants and food products, such as honey and lactic acid bacteria-fermented food, and has a broad-spectrum antibacterial effect (Ning et al., 2017). The researched area found that PLA can also inhibit the formation of bacterial biofilms, such as *Listeria monocytogenes* (*L. monocytogenes*) and *E. cloacae* (Liu et al., 2017a,b). In the study about *E. faecalis* (Liu et al., 2020b), it was found that the number of bacteria adhering to polyethylene plate and stainless-steel plates decreased significantly after PLA treatment, and that the ability of adhesive bacteria to produce EPSs was also inhibited. Reverse-transcription polymerase chain reaction confirmed that PLA significantly inhibited the expression of EPA polysaccharide synthesis genes, suggesting that PLA could reduce the bacterial adhesion rate and inhibit early stage biofilms development by reducing extracellular polysaccharide synthesis.

#### Probiotics

*Lactobacillus* is one of the probiotic species and can play a potent role in promoting body development, stimulating the immune system, and has an inhibitory effect on the formation of *P. aeruginosa* and *Salmonella* biofilms (Shokri et al., 2018; Merino et al., 2019). In the study with *S. aureus* (Melo et al., 2016), it was observed that treatment with *Lactobacillus* significantly increased the expression of *icaR* and decreased the expression of *icaA*, leading to the inhibition of the synthesis of PIA and affecting the adhesion between bacteria and early stage biofilms formation. *Lactobacillus salivarius* and *Lactobacillus kefiranofaciens* can inhibit the expression levels of *gtfBCD* and *ftf*, respectively, thereby reducing the synthesis of exopolysaccharides, hindering initial adhesion, and inhibiting early stage biofilms formation of *Streptococcus mutans* (*S. mutans*) (Wu et al., 2015; Jeong et al., 2018).

### Reducing Extracellular Proteins Production

In general, only proteins that play a structural role have been clearly characterized, and matrix proteins are poorly defined and understood (Tseng et al., 2018). However, matrix proteins have been suggested to promote bacterial adhesion and early stage biofilms accumulation (Matsumoto-Nakano, 2018). For example, in *S. aureus*, the biofilm-associated protein (BAP) promotes initial attachment and cell-to-cell interaction through mechanisms independent of extracellular polysaccharides. When BAP is inhibited, the abilities of primary attachment and biofilms formation are significantly reduced (Cucarella et al., 2001). Therefore, reducing the production of extracellular proteins is one of the effective methods to control early stage biofilms formation, and D-amino acids and rhamnolipid are believed to exert biofilms inhibition by affecting extracellular proteins.

#### D-Amino Acids

D-amino acids, also called unnatural amino acids, are enantiomers with L-amino acids and naturally exist in organisms. Researchers first demonstrated that 6 mg/L D-tyr could remove biofilms from nylon membrane surfaces (Xu and Liu, 2011). Subsequent studies showed that D-amino acids have an inhibitory effect on the formation of biofilms. For example, a mixture of D-leu and D-try can inhibit the formation of *S. aureus* and *P. aeruginosa* biofilms, and a mixture of D-pro and D-ala can inhibit the formation of *S. epidermidis* biofilms (Kolodkin-Gal et al., 2010; Ramón-Peréz et al., 2014).

At experimental concentrations, the addition of exogenous D-amino acids has no effect on bacterial growth, but can significantly inhibit the formation of biofilms (Li et al., 2015; She et al., 2015), suggesting that the inhibition of bacterial growth is not the mechanism by which D-amino acids inhibit the formation of biofilms. D-amino acids inhibit biofilms development by reducing initial adhesion.

In *P. aeruginosa* and *S. aureus*, the production of extracellular proteins was significantly reduced after D-amino acids treatment, but had no effect on exopolysaccharides (Hochbaum et al., 2011; Yu C. et al., 2016). D-amino acids are substituted for L-amino acids in a protein (Pundir et al., 2018), and the protein undergoes changes in structure, making the protein minimally secreted. This finding suggests that the change in extracellular proteins may be the cause of D-amino acids inhibition of adhesion. Thermodynamic research reported that with the addition of D-tyr, Lewis acid–base interactions are more affected than the overall non-specific interactions (Xing et al., 2015). The Lewis acid-base force is closely related to hydrophilicity, and the change in EPS has a negative effect on the water contact angle (Sheng and Yu, 2006). The decrease in the protein/polysaccharide ratio is likely to be the cause of increased hydrophilicity, which further indicates that D-amino acids inhibit adhesion and biofilms formation by reducing extracellular protein synthesis.

Peptidoglycan (PG), the major component of the cell wall, is composed of repeating units of GlcNAc and *N*-acetylmuramic acid (MurNAc). A short peptide chain terminating in D-alanine is attached to the lactoyl group of MurNAc, and researchers revealed that exogenous D-amino acids could replace D-alanine to integrate into PG (Lam et al., 2009), changing the composition and architecture of PG. The modification of PG affects the cell wall, and the cell wall is one of the important factors mediating initial adhesion, indicating that D-amino acids may inhibit initial adhesion by affecting the cell wall.

#### Rhamnolipids

In recent years, rhamnolipids derived from *P. aeruginosa* have emerged as an important group of biosurfactants with several applications, and are considered a substitute for traditional antibacterial agents. Current studies found that rhamnolipids disrupted many bacterial biofilms, and the exogenous addition of rhamnolipids could impede initial adhesion (Dusane et al., 2010). The study on *P. aeruginosa* biofilm showed that after rhamnolipids treatment, the reduction in protein concentrations was more than twice the reduction in carbohydrate concentrations (Kim et al., 2015). This result was similar to the later finding that strains with high protein content in the biofilm matrix were more susceptible to surfactants than strains with carbohydrate-based matrix (Silva et al., 2017), indicating that rhamnolipids can have a selective interaction of protein to reduce protein contents and affect the early adhesion of bacteria. Rhamnolipids can affect the swarming motility in addition to reducing protein. The swarming motility is a cell density-dependent, flagella-driven process that enables bacteria to move toward favorable environments and adhere. Studies demonstrated that when rhamnolipids were absent, bacteria lost their ability to swarm, which can be supplemented by providing exogenous rhamnolipids (Caiazza et al., 2005; Nickzad et al., 2015). Therefore, reducing the rhamnolipids content in the bacterial population to hinder swarming motility is also an effective method to inhibit early adhesion. Adding rhamnolipids reduces the protein content but promotes swarming motility. Thus, an in-depth study is needed.

#### Others

Proteases, which specifically hydrolyze extracellular proteins, may have great ability to control initial adhesion. When *bap*-positive *S. aureus* were treated with proteinase K, the number of bacteria attached to the microtiter plate at the primary stage was significantly reduced (Kumar Shukla and Rao, 2013), indicating that exogenous proteases destroyed the physical stability of the EPS and hampered early adhesion. Later studies showed that protease K can hydrolyze extracellular proteins and reduce the eDNA content (Shukla and Rao, 2017). Papain and trypsin can digest the fimbrial proteins FimP and FimA (Mugita et al., 2017). eDNA and fimbriae also play an important role in initial adhesion, which further suggests that proteases can be an anti-adhesive agent to affect the early stage biofilms formation.

Curcumin is a hydrophobic polyphenolic substance extracted from the dried rhizomes of Turmeric, a plant of the genus *Curcuma*, and is recognized as a safe food additive. In addition to being a natural food coloring, curcumin is found as an inhibitor of sortase A activity in *S. aureus* (Hu et al., 2013b). Curcumin can inhibit surface protein production or separate protein from cell walls, thereby reducing the initial adhesion and inhibiting early stage biofilms formation (Park et al., 2005; Hu et al., 2013a).

### Reducing eDNA Production

The content of eDNA is significantly less than those of proteins and exopolysaccharides, and related studies are few. However, eDNA is generally believed to promote initial bacterial adhesion and surface aggregation (Jakubovics and Burgess, 2015). eDNA promotes the adhesion of bacteria to biotic and abiotic surfaces, and its essence may have the following points. First, irreversible adhesion is mediated by Lewis acid-base forces, and thermodynamic analysis showed that the presence of eDNA promotes the acid-base interaction (Das et al., 2010), which is more conducive to the attachment of bacteria on the surface, and has a remarkable promotion effect on the formation of biofilms. Second, eDNA does not exist independently in EPSs and interacts with other biomolecules in the biofilm matrix (Okshevsky et al., 2015). Maintaining the integrity of the EPS structure and facilitating the adhesion between bacteria and surface are important. Researchers found that the removal of eDNA also results in the reduction of exopolysaccharides and proteins (Yu et al., 2018). Third, the presence of eDNA widens the adhesion distance, facilitating bacteria to begin irreversible adhesion at a large distance scale (Okshevsky and Meyer, 2015), which remarkably promotes the formation of early stage biofilms. Therefore, reducing eDNA production is also an effective method to inhibit initial adhesion, and current studies confirmed that DNase and reserpine exert early stage biofilms inhibition by affecting eDNA.

#### DNase

At present, DNase is the most in-depth studied strategy of removing eDNA, and can be used to decompose eDNA to reduce bacterial adhesion and control the formation of early stage biofilms. Studies showed that after treating *L. monocytogenes* (Harmsen et al., 2010), *Burkholderia pseudomallei* (Pakkulnan et al., 2019) and *S. aureus* (Parastan et al., 2020) with DNase, the bacteria attached to the surface were significantly reduced, and the formation of biofilms were inhibited. The first functional DNase I coating has reduced bacterial adhesion by 95–99% (Swartjes et al., 2013). However, DNase can easily remove young biofilms and partially inhibit middle-stage biofilms, but has no effect on mature biofilms (Okshevsky et al., 2015; Schlafer et al., 2017).

#### Others

Reserpine, a drug used to treat hypertension and psychosis, has been recently found to inhibit the formation of *S. aureus* biofilms because it has the same active site as the AtlE protein ligand (Parai et al., 2020). Reserpine competitively inhibits the binding of ligands to AtlE protein, thereby reducing the synthesis and secretion of eDNA (Kerckhoven et al., 2016), resulting in decreased adhesion and inhibiting biofilms formation.

Totarol, a diterpene compound isolated from the totarol tree, has remarkable antibacterial and antioxidant activities. In the study with *S. aureus* biofilms (Guo et al., 2015), the expression levels of the *icaA* gene encoding exopolysaccharide synthesis and the *cidA* gene encoding eDNA synthesis decreased significantly after the action of totarol, and the expression amount of *cidA* decreased more, indicating that totarol reduces the synthesis of eDNA to inhibit early adhesion.

Researchers found that a biosurfactant can significantly down-regulate the expression of *dltB* and *cidA* genes in *S. aureus* and that the *dltB* operon promotes bacterial adhesion (Yan et al., 2019). Among the two adhesion-related genes, *cidA* expression is more significantly affected than *dltB* expression, suggesting that this biosurfactant reduces adhesion by inhibiting eDNA.

## Inhibition of the QS System

The QS system confers bacteria as a community to approach the surface in a consistent manner and increase the secretion of EPS, making the attachment firmly, which is beneficial to the formation of early stage biofilms. According to the chemical types of signal molecules, the QS system can be roughly divided into three types (Wu et al., 2020): (1) *N*-acyl-homoserines (AHLs)-mediated QS system, which exists in Gram-negative bacteria; (2) auto-inducing peptide (AIP)-mediated QS system, which exists in Gram-positive bacteria; and (3) autoinducer 2 (AI-2)-mediated QS system, which exists in Gram-negative and Gram-positive bacteria. Studies found a QS system mediated by the Pseudomonas Quinolone Signal (PQS) in *P. aeruginosa* (Li et al., 2020).

**FIGURE 3:**
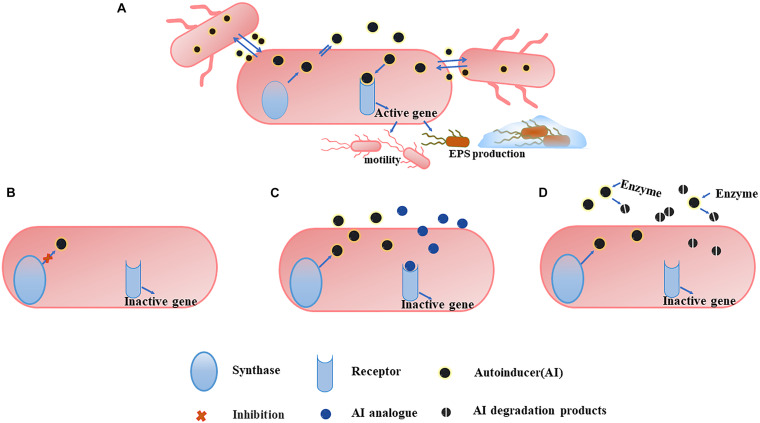
An overview of QS system **(A)** and the three main strategies to combat it. **(B)** Blocking the generation of AI. The inhibition of some of synthase can affect the production of more than one signal. **(C)** Preventing AI from binding to the corresponding receptors. The use of AI analogs can competitively inhibit the binding of AI to the receptor, and the analogs themselves have no pharmacological activity, so they cannot activate the corresponding gene expression and the QS mechanism is ceased. **(D)** Degrading the generated AI. Degrading the AI with the corresponding enzyme makes it unable to reach the threshold concentration and the degradation products cannot bind to the receptor, inhibiting the QS system.

Given the important role of QS in early stage biofilms development, the mode targeting at QS has also become an alternative approach to combat undesirable microorganisms. By acting on different steps of the signaling cascade, QS inhibitors can be divided into three categories (Kalia et al., 2019), (1) blocking the generation of signal molecules, (2) preventing the signal molecules from binding to the corresponding receptors, and (3) degrading the generated signal molecules (Figure 3). Different QS inhibitors are described as follows.

### Blocking the Generation of Signal Molecules

Coumarins, a group of heterocyclic compounds present in many natural plants, have a wide range of biological activities such as antiviral and antioxidation properties, and can be used as QS inhibitors to affect the formation of biofilms such as *E. coli*, *P. aeruginosa*, and *S. aureus* (D’Almeida et al., 2017; Das et al., 2018; Reen et al., 2018). The study about *P. aeruginosa* found that the expression levels of *lasI*, *rhlI*, and *pqsBCH* that encoding signal molecules synthesis was significantly downregulated after the action of 2 mM coumarin, whereas those of receptor genes *lasR* and *pqsR* remained unchanged (Zhang et al., 2018). As a result, the syntheses of AHL molecules and PQS was hindered, affecting the QS system and reducing the formation of biofilms. Eugenol has similar mechanism of action with coumarins. After treatment with eugenol and its nanoemulsion at a concentration of 0.2 mg/mL, the expression levels of QS synthase genes *lasI* and *rhlI* were inhibited. The inhibition effect led to a decrease in the production of 3-oxo-C12-HSL and C4-HSL, which affected swarming motility and biofilms formation of *P. aeruginosa* (Lou et al., 2019).

Salicylic acid, the bulk drug used to synthesize aspirin, is also a strong inhibitor of AHL and interferes with the production of AHL by binding to the substrates needed for its production (Shaaban et al., 2019). *Trans*-cinnamaldehyde interacts with the substrate binding site of LasI to inhibit AHL production (Chang et al., 2014; Shaaban et al., 2019). Triclosan reduces the production of AHL and inhibits the QS system by inhibiting the enoyl-acyl carrier protein reductase which is an essential intermediate in AHL biosynthesis (Basavaraju et al., 2016).

### Preventing Signaling Molecules From Binding to Corresponding Receptors

Furan compounds are originally discovered in the red marine alga *Delisea pulchra*. Given their similarity in structure with AHLs, they can replace AHLs to bind with corresponding LasR receptors and inhibit the QS system (Kim et al., 2012; Kalia, 2013). This competitive inhibitory effect is dependent on dose. Thus, the side effects of high doses should be considered when developing furan compounds as QS inhibitors. (5Z)-4-bromo-5-(bromomethylene)-3-butyl-2(5H)-furanone has been shown to act as antagonist of AHL and AI-2 QS signals in many organisms (Kalia et al., 2019). At concentrations of 2.0 and 4.0 μg/mL, furanone C-30 could inhibit exopolysaccharide genes *ftf* and *gtfB* and extracellular protein gene *gbpB* in the biofilm of *S. mutans* without affecting the growth of planktonic bacteria (He et al., 2012), thereby reducing the initial adhesion and inhibiting the formation of biofilms. Chlorogenic acid (CA) (Deryabin et al., 2019), flavonoids (Liu et al., 2017d; Abinaya and Gayathri, 2019), and cinnamaldehyde (Li et al., 2018) have similar mechanisms of action with furan compounds. After the action of CA, the production of rhamnolipid was inhibited (Wang et al., 2019), further affecting the early stage biofilms formation of *P. aeruginosa*.

In addition, the apolipoprotein B can prevent AIP1 from binding to the receptor and inhibit the QS system of methicillin-resistant *S. aureus* (Peterson et al., 2008).

### Degrading Generated Signal Molecules

In the QS system, it is critical for AHL molecules to reach a threshold concentration. Therefore, if AHLs can be degraded to make it unable to reach the threshold concentration, the formation of biofilms is remarkably weakened. According to the chemical structure of AHLs, three action sites, namely, lactonase site, acyltransferase site, and oxidoreductase site, can decompose AHLs (Huang et al., 2016). The degradation of AHL is largely regulated by AHL acyltransferase and AHL lactonase, which hydrolyze the bond and HSL ring, respectively (Brackman and Coenye, 2015; Haque et al., 2018), and degrade AHLs to inhibit the QS system.

## Targeting c-di-GMP Signaling

**FIGURE 4:**
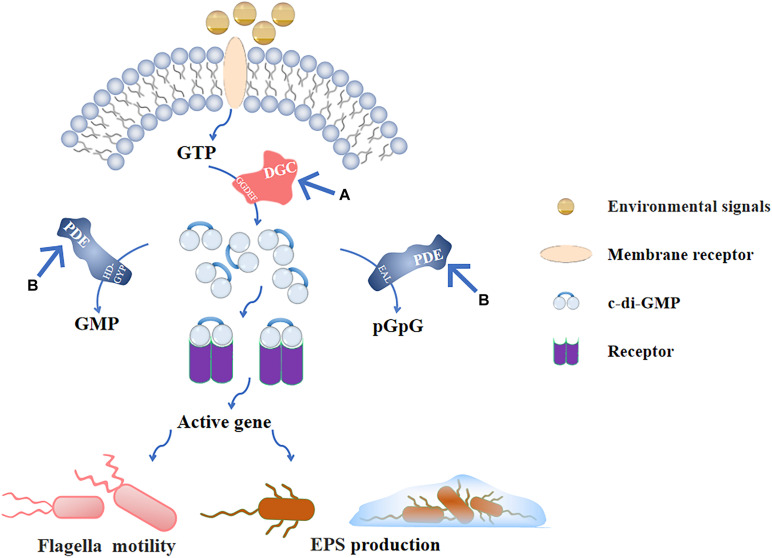
C-di-GMP signaling pathway and **(A)**, **(B)** are the main inhibitory modes. After the environmental signals are combined with the receptor on the membrane, GTP is produced in the cell. GTP is catalyzed by the GGDEF domain in DGC to form c-di-GMP, and produced c-di-GMP binds to the intracellular receptor to activate the corresponding gene expression and regulate flagellar movement and EPS production; PDE also exists in the cell, and the EAL domain and HD-GYP domain can, respectively, hydrolyze c-di-GMP into pGpG and GMP to inactivate it. **(A)** Inhibiting the activity of DGC prevents GTP from producing c-di-GMP, so that downstream genes cannot be regulated. **(B)** Stimulating the activity of PDE to decrease the intracellular c-di-GMP levels that is negatively affects the formation of early stage biofilms.

The c-di-GMP signaling plays critical roles during the biofilms formation. Flagellar motility and EPS secretion, which are partly regulated by c-di-GMP signaling and facilitate early stage biofilms development. Thus, blocking c-di-GMP signal transduction is important to inhibit the early stage biofilms. The c-di-GMP-mediated signaling pathway consists of four parts (Boyd and O’Toole, 2012): (1) DGCs that promote the synthesis of c-di-GMP, (2) effector molecules combined with c-di-GMP, (3) downstream targets regulated by effector molecules, and (4) PDEs that degrade c-di-GMP. Among them, the synthesis and degradation of c-di-GMP by DGCs and PDEs are the core of the whole signaling pathway (Figure 4). The following describes the corresponding methods based on these two targets.

### Targeting DGCs

Controlling endogenous synthesis of c-di-GMP is more efficient than directly acting on the downstream targets of c-di-GMP. A glycosylated triterpenoid saponin purified from extracts of garden pea is identified as the first effective inhibitor of DGC, and Papulacandin B and *N*-(4-anilinophenyl) benzamide are subsequently demonstrated as inhibitors (Opoku-Temeng and Sintim, 2017). Considering that the catalytic domain of DGCs is similar to the catalytic domain of mammalian adenylyl cyclases, and 2′(3′)-*O*-(*N*-methylanthraniloyl) (MANT)- and 2′,3′-*O*-(2,4,6-trinitrophenyl) (TNP)-substituted nucleotides are potent adenylyl and guanylyl cyclase inhibitors, researchers tested various MANT- and TNP- substituted nucleotides and found that MANT-GTP, MANT-GTPγS, and TNP-GTP acted as potent inhibitors against *E. coli* DGCs (Spangler et al., 2011).

### Targeting PDEs

Stimulating the activity of PDE keeps the intracellular c-di-GMP at a low concentration, which has a negative effect on biofilms formation. Endogenously produced nitric oxide (NO) is a signaling molecule that reduces the concentration of c-di-GMP in the population by stimulating the activity of PDE (Vuotto and Donelli, 2019). In clinical trials, the inhalation of a low dose of NO (10 ppm) significantly reduced *P. aeruginosa* biofilm aggregation in cystic fibrosis patients (Howlin et al., 2017). The application of NO-releasing chitosan dressings to *S. aureus*-infected wounds in mice could enhance biofilm dispersal (Choi et al., 2020). Here, we emphasize that the low concentration of NO can trigger biofilm dispersal because a high concentration can promote the biofilm formation of some bacteria, such as *P. aeruginosa* and *Nitrosomonas europaea* (Arora et al., 2015). This phenomenon may be because a high concentration of NO causes anaerobic environments, and forming biofilms in anaerobic environments can be used as a stress defense mechanism (Cutruzzolà and Frankenberg-Dinkel, 2016). Therefore, the critical concentration should be determined before using NO as an antibacterial agent.

## Conclusion

Researches about biofilms has basically begun in the 1970s (Gebreyohannes et al., 2019). Once the biofilms mature, they are difficult to eradicate due to the EPS barrier function and the efflux pump system, causing serious harm. Thus, intervening in the early stage of biofilms formation can be a solution. The inhibition of early stage biofilms formation can be accomplished by affecting the adhesion of bacteria. Reducing bacterial adhesion can be divided into two points. One is to prevent bacteria from approaching the surface, which is mainly by changing the surface properties or reducing the movement of bacteria, and the other is to reduce the production of EPSs.

Researches on the inhibition of biofilm formation based on hydrophilic coatings changing the properties of the surface and antimicrobial coatings killing the attached bacteria have been relatively mature, but these methods are suitable for biofilms formation of related equipment and implants. Using molecular drugs to inhibit early stage biofilms formation from internal mechanisms is a research hotspot. The movement ability and EPS secretion of bacteria are important factors that affect the formation of early stage biofilms. At the same time, the QS system and c-di-GMP signaling pathway regulate these two behaviors, so early stage biofilms inhibitors can be divided into: (1) reducing bacterial movement ability, (2) inhibiting EPS production, (3) interfering with QS system, and (4) destroying c-di-GMP signal pathway. Inhibitors of the same type can also exert corresponding effects through different pathways, for example, with the same extracellular protein inhibitors, D-amino acids can reduce protein production, rhamnolipids can bind to the protein, and proteases can hydrolyze the protein. Early stage biofilms are fragile, have no 3D structures yet, and are relatively easy to be removed. Early stage biofilms treatments combined with antibiotics have the potential to achieve better therapeutic effects compared to monotherapy, as exemplified by Zhang et al. (2015) and Liu et al. (2017c). Thus, early intervention is necessary.

To date, efforts have already been made in the biofilms field, but current researches on the early inhibition of biofilms have not achieved effective breakthroughs due to two main reasons. First, current techniques for detecting early stage biofilms infections are immature. When infections caused by biofilms are diagnosed, they often develop to the mature biofilm, and this presents a substantial problem in having the opportunity to treat infections at the early stage biofilm progression in the first place. Second, clinical studies are lacking. The differences between *in vitro* and *in vivo* experiments are large, and current *in vitro* assays cannot effectively guarantee the outcomes in clinical practices. *In vitro* experiments can only preliminarily prove that drugs have therapeutic effects. When the body is infected with biofilms, it will undergo complex changes, which cannot be effectively simulated by *in vitro* experiments. These phenomena hinder the progress toward the further application for some types of molecules. Therefore, future researches should focus on the establishment of early stage biofilms infection models, *in vivo* experiments, and improvement of detection technology.

## Author Contributions

JF drafted the manuscript. YZ and SL were responsible for preparing the tables and figures in the manuscript. WZ, JL, HL, FX, HT, GP, LZ, and SC reviewed the manuscript. HF conceptualized the content of the manuscript. All authors contributed to the article and approved the submitted version.

## Conflict of Interest

The authors declare that the research was conducted in the absence of any commercial or financial relationships that could be construed as a potential conflict of interest.
